# Irisin reshapes bone metabolic homeostasis to delay age-related osteoporosis by regulating the multipotent differentiation of BMSCs via Wnt pathway

**DOI:** 10.3389/fmolb.2024.1524978

**Published:** 2025-01-07

**Authors:** Shangman Xing, Yifan Ma, Bing Song, Min Bai, Kexin Wang, Wenjing Song, Tingting Cao, Chao Guo, Yanying Zhang, Zhandong Wang, Yongfeng Wang

**Affiliations:** ^1^ The First Clinical Medical College, Gansu University of Chinese Medicine, Lanzhou, China; ^2^ Division of Cancer Biology, Laboratory Animal Center, Fourth Military Medical University, Xi’an, China; ^3^ Medicine Research and Experimental center, Gansu University of Chinese Medicine, Lanzhou, China; ^4^ Ningxia Medical University College of Traditional Chinese Medicine, Yinchuan, China; ^5^ Clinical College of Integrated Traditional Chinese and Western Medicine, Gansu University of Chinese Medicine, Lanzhou, China; ^6^ Gansu Medical University School of Basic Medicine, Pingliang, China

**Keywords:** aging, BMSCs, irisin, age-related osteoporosis, bone-fat balance

## Abstract

**Introduction:**

Bone aging is linked to changes in the lineage differentiation of bone marrow stem cells (BMSCs), which show a heightened tendency to differentiate into adipocytes instead of osteoblasts. The therapeutic potential of irisin in addressing age-related diseases has garnered significant attention. More significantly, irisin has the capacity to enhance bone mass recovery and sustain overall bone health. Its mechanism of action in preventing osteoporosis has generated considerable interest within the research community. Nonetheless, the targeting effect of irisin on age-related osteoporosis and its underlying molecular biological mechanisms remain unclear.

**Methods:**

The specific role of irisin in osteogenic-adipogenic differentiation in young or aging BMSCs was evaluated by multiple cells staining and quantitative real-time PCR (RT-qPCR) analysis. RNA-seq and protein Western blotting excavated and validated the key pathway by which irisin influences the fate determination of aging BMSCs. The macroscopic and microscopic changes of bone tissue in aging mice were examined using Micro-computed tomography (Micro-CT) and morphological staining.

**Results:**

It was noted that irisin affected the multilineage differentiation of BMSCs in a manner dependent on the dosage. Simultaneously, the Wnt signaling pathway might be a crucial mechanism through which irisin sustains the bone-fat balance in aging BMSCs and mitigates the decline in pluripotency. *In vivo*, irisin reduced bone marrow fat deposition in aging mice and effectively alleviating the occurrence of bone loss.

**Conclusion:**

Irisin mediates the Wnt signaling pathway, thereby influencing the fate determination of BMSCs. In addition, it is essential for preserving metabolic equilibrium in the bone marrow microenvironment and significantly contributes to overall bone health. The findings provide new evidence for the use of iris extract in the treatment of age-related osteoporosis.

## 1 Introduction

Age-related osteoporosis constitutes a metabolic bone disorder marked by systemic bone loss, prominently presented as decreased bone mineral density, disruption of bone microstructure, multiple fractures, and augmented accumulation of bone marrow fat (Ge et al., 2017; Reyes Fernandez et al., 2016; Tu et al., 2018). With the acceleration of the aging process, it is projected that by 2050, individuals aged 60 and above will constitute over 20% of the global population (Sipos et al., 2009). This demographic transformation has emerged as a prominent public health issue that attracts worldwide attention (Compston et al., 2019; Qadir et al., 2020). Age-related osteoporosis is correlated with pathological alterations induced by aging, encompassing inflammatory procedures, increased parathyroid hormone levels, deficiencies in calcium and vitamin D, or dysfunction of osteoblasts (Tu et al., 2018). Currently, the pharmacological treatments for age-related osteoporosis, such as bisphosphonates and denosumab, are associated with various side effects including ocular tremors, hypertension, headaches, and sensory imbalances. These adverse effects can severely impact the quality of life and prognosis of older adults (Luhmann et al., 2012). Therefore, it has become increasingly urgent to further explore the pathogenesis of age-related osteoporosis and to identify safe and effective novel anti-osteoporotic drugs.

Bone marrow mesenchymal stem cells (BMSCs) are multipotent stem cells originating from the mesoderm, possessing the capability for multidirectional differentiation (Hoang et al., 2022). Under normal circumstances, BMSCs readily differentiate into osteoblasts to maintain bone homeostasis. As age increases and the duration of *in vitro* culture extends, the self-renewal capacity of BMSCs diminishes (Galipeau and Sensébé, 2018). Consequently, these cells tend to differentiate into adipocytes rather than osteoblasts (Pittenger et al., 1999). This shift promotes the interconversion between fat and bone mass within the bone marrow cavity, leading to an imbalance between bone-fat (Ouyang et al., 2022). Therefore, BMSCs exert a crucial role in the endocrine and paracrine interaction and equilibrium between bone-fat (Reyes Fernandez et al., 2016; Pereira et al., 2020), and it is worthy of exploring targeted therapeutic drugs and their mechanisms of action.

Irisin is a novel myokine secreted during skeletal muscle movement, which is derived from fibronectin type III domain containing protein 5 (FNDC5) (Boström et al., 2012; Metwally et al., 2019). Clinical studies have demonstrated that serum irisin expression is markedly reduced in middle-aged and elderly men and is positively associated with bone mineral density (BMD) (Zhang et al., 2020; Zhou et al., 2019), indicating that irisin might serve as a novel biomarker for age-related osteoporosis. It has been documented that irisin is capable of interacting with αV integrin receptors 5 (αV/β5) to activate the bone morphogenetic protein (BMP)/small mother against decapentaplegic (SMAD) pathway and stimulate autophagy, thereby facilitating the osteogenic differentiation of BMSCs (Chen et al., 2020; Xue et al., 2022). Concurrently, functioning as an adipokine, irisin is capable of promoting the brown remodeling of white adipose tissue and suppressing the formation of adipose tissue (Xiong et al., 2018), which is mediated through facilitating the augmentation of Wnt signaling and its downstream transcription factors (Zhu and Fan, 2022). It is widely acknowledged that the Wnt signaling cascade determines the differentiation direction of BMSCs and governs adipogenesis (Ma et al., 2019). Therefore, irisin may serve as a pivotal target for the regulation of fat and bone metabolism. It is anticipated that further exploration of its underlying mechanisms and potential applications will advance the treatment of age-related osteoporosis.

The study has discovered that irisin is associated with the alterations in multidirectional differentiation potential and the aging process of BMSCs. The transcriptional expression of β-catenin was reduced in aging BMSCs. Meanwhile, irisin enhanced the expression of β-catenin proteins, which is involved in the regulation of multi-differentiation of BMSCs. Moreover, in the aging mouse model, it has been demonstrated that irisin promotes skeletal bone health. This study highlights that irisin can modulate the balance between bone and fat to sustain bone metabolic homeostasis, presenting it as a potential therapeutic candidate for age-related osteoporosis.

## 2 Materials and methods

### 2.1 Animal experiment protocol

Male C57BL/6 mice (2 months old, average weight: 20 ± 2 g and 15 months old, average weight: 30 ± 5 g) were purchased from Beijing Weitonglihua Co., Ltd. (NO.1367569). They were housed in the SPF-free animal facility of the Laboratory Animal Center at Gansu University of Traditional Chinese Medicine. The mice had free access to food and water, lived in an environment with a temperature range of 18°C–22°C, experienced a 12 h light/dark cycle, and were maintained at a humidity level between 40% and 70%. Prior to the formal experiment, they underwent a one-week acclimatization period.

We randomly divided the mice into four groups based on age: the Young-Vehicle group, the Aged-Vehicle group, the Aged-L-irisin group (25 mg/kg, L-irisin), and the Aged-H-irisin group (100 mg/kg, H-irisin). The irisin dosage was based on previous literature (Storlino et al., 2023). Vehicle or irisin was administered once a week via the caudal vein for 4 weeks. After 4 weeks, the mice were euthanized with isoflurane (RWD, Lot.21121901, Shenzhen, China) and samples were collected.

All experimental animal protocols were approved by the Experimental Animal Ethics Committee of Gansu University of Traditional Chinese Medicine (Ethics Approval number: 2023–560, approval date: 2023.7.15). All animal studies were performed following the protocols established by the Institutional Animal Care and Use Committee (IACUC) and adhering to the ARRIVE guidelines, in alignment with the 3R Principles.

### 2.2 Primary cell extraction and culture

Bone marrow was carefully flushed from the femur and tibia of C57BL/6J mice (2 months old and 15 months old) and subsequently cultured in complete growth medium, consisting of 90% DMEM/F12 (Vivacell, Lot.2404039, Shanghai, China), 10% Fetal Bovine Serum (YEASEN, Lot NO.504040622, Shanghai, China), and 1% Pen-Strep Solution (Vivacell, Lot. 2,311,056, Shanghai, China), under controlled conditions at 37°C and 5% CO₂. The culture medium was refreshed every 1–2 days to maintain optimal conditions. Once the cell density reached approximately 90%, the cells were passaged, and subsequent passages (typically 1-2 generations) were collected for experimental use. Cell surface markers CD29 (Biolegend, Lot. B372474, California, U.S.), CD34 (Liankebio, Lot. A404448, Hangzhou, China), and CD44 (Abcam, Lot. GR3398810-7, London, United Kingdom) were then analyzed and confirmed via flow cytometry.

At approximately 80% cell confluence, the cells were induced to differentiate: complete osteogenic medium was used to promote bone formation, while lipid induction medium was employed to stimulate lipogenesis.

### 2.3 The CCK-8 experiment

A suspension of BMSCs with a density of 1×10^4^ cells per well was carefully inoculated into each well of a 96-well plate. BMSCs were then cultured in an incubator under optimal conditions for a period of 24 h to allow for proper adhesion and growth. Following this initial incubation period, the cells were subjected to an experimental intervention involving varying concentrations (0.1, 1, 5, 10, and 20 ng/mL) of irisin (MCE, Lot.190040, NJ, US). To assess cell viability post-treatment, a CCK-8 solution (Sunview, Lot. DCM2188, Shenzhen, China) was introduced into each well, and the absorbance was measured at 450 nm using an enzymatic microplate reader. This absorbance data provided a quantitative evaluation of cell viability in response to the irisin treatment.

### 2.4 Alkaline phosphatase staining

Seven days after the induction of osteogenesis, the cells were rinsed 2–3 times with PBS and then fixed in a 4% histopathological fixation solution for a duration of 15 min. Staining was then performed utilizing the pluripotent stem cell alkaline phosphatase (ALP) chromogenic kit (Beyotime, Lot.080423230831, Shanghai, China). Imaging was conducted with a live cell workstation, and quantitative analysis was carried out using ImageJ to calculate the percentage of the positive area.

### 2.5 Alizarin red staining

On the 14th day after osteogenesis was induced, the samples underwent 2-3 washes with PBS and were then fixed in a 4% paraformaldehyde solution for 15 min. Alizarin red (ARS) staining (AccuRef Scientific, Lot. A23E201, Xian, China) was then performed, and imaging was conducted using a live-cell workstation. The positive area and percentage were subsequently analyzed using ImageJ software.

### 2.6 Oil red O staining

After 21 days of lipid induction, the cells were washed once by PBS, fixed and stained with reference to the oil red O(ORO) staining kit (Beyotime, Lot.080223230815, Shanghai, China), and the lipid droplets were observed and photographed under the microscope 3 h later. The analysis of positive area was conducted using ImageJ soft-ware.

### 2.7 β-galactosidase (SA-β-Gal) staining

After culturing young or aged BMSCs for 7 days, the cells were fixed with 4% paraformaldehyde. Following the protocol provided in the kit (Beyotime, Lot.070623231025, Shanghai, China), β-galactosidase staining was performed, and images were captured after a 24-h incubation period. The analysis of positive area was conducted using ImageJ software.

### 2.8 Quantitative real-time PCR (qPCR)

Total RNA was isolated from cells and bone tissues using a cell RNA extraction kit (TIANGEN Lot. W9727, Beijing, China), and RNA concentrations were quantified using a spectrophotometer. Complementary DNA (cDNA) was synthesized through reverse transcription employing a commercially available reverse transcription kit (Yeason, Lot. H6326010, Shanghai, China). qPCR was subsequently performed with SYBR Green dye (Yeason, Lot. H5310690, Shanghai, China), utilizing β-actin as an internal reference for normalization. Relative gene expression levels were determined based on established methodologies cited in previous publications. Primer sequences are detailed in Table 1.

**TABLE 1:** The primer sequences of mice.

Genes	Primer Forward (5’ –3′)	Primer Reverse (3′–5′)
β-actin	CATCCGTAAAGACCTCTATGCCAAC	ATGGAGCCACCGATCCACA
ALP	TCCGTGGGTCGGATTCCT	GCCGGCCCAAGAGAGAA
Runx2	CAAGAAGGCTCTGGCGTTTA	TGCAGCCTTAAATGACTCGG
PPAR-γ	GCTGACCCAATGGTTGCTGA	TTCATGAGGCCTGTTGTAGAGCTG
C/EBPα	TTGAAGCACAATCGATCCATCC	TGCACACTGCCATTGCACA
ATF4	CACCGGAAATTCGTCAACGAG	TGTGGCGTTAGAGATCGTCCTAAAG
β-catenin	GCTGCTGTCCTATTCCGAATGTC	GGCACCAATGTCCAGTCCAA

### 2.9 Transcriptomics and bioinformatics analysis of RNA sequence

BMSCs from young and senescent C57BL/6J mice were extracted and cultured. Irisin (5 ng/mL) was applied to senescent BMSCs in three groups, with three biological replicates per group. Transcriptomic assays were performed by Nomi Metabolism (Nuomi, Suzhou, China). Total RNA was qualitatively assessed, and a cDNA library was constructed before RNA sequencing was carried out. After the extraction, purification, and library construction of RNA, Next-Generation Sequencing (NGS) technology was employed to conduct paired-end (PE) sequencing of these libraries utilizing the Illumina sequencing platform. Differentially expressed genes (DEGs) were deter-mined by using the corrected thresholds of P ≤ 0.05 and log2 (fold change) ≥1 for both upregulated and downregulated genes. The data packages facilitated the differentiation between the samples from young and old ones and thus revealed the changes in RNA expression related to aging and treatment conditions. The DEG, having the lowest adjusted P-value, was used for the heat map analysis. Gene set enrichment analysis (GSEA) was carried out with the GSEA v4.3.0 program on the expressed genes and overrepresented categories were screened by Gene Ontology and the Kyoto Encyclopedia of Genes and Genomes (KEGG). Several types of differentially variable shear events were analyzed, and their percentages were computed |ΔINC| > 0.05, FDR ≤0.01. The sequencing data has been deposited in the National Center for Biotechnology In-formation (NCBI) database (PRJNA1182913).

### 2.10 Micro-CT analysis

The femur and vertebrae of the mice were collected and fixed in 4% paraformaldehyde for a duration of 48 h. Micro-CT imaging technology (Caliper, BR700000035-01, Waltham, US) was employed for scanning and analysis. During the scanning process, the specimens were positioned within the scanning chamber with the long axis of the bone tissue oriented perpendicular to the X-ray beam. Subsequently, three-dimensional reconstruction was carried out using analytical software provided by the system. For analysis of the femur, approximately 5% of the area beneath the growth plate was designated as the region of interest, while vertebral bodies spanning L4-S1 were selected for further examination. Key parameters including bone mineral density (BMD), bone volume fraction (BV/TV), surface fraction (BS/TV), trabecular thickness (Tb.Th), trabecular number (Tb.N), and trabecular separation distance (Tb.Sp) were calculated over this selected scan range encompassing 100 sections.

### 2.11 Histomorphometry

The fixed bone was sliced by paraffin embedding and dehydrated with xylene, anhydrous ethanol and 75% alcohol. Then dye with hematoxylin dye solution, rinse with running water, add H&E dye solution (Service bio, Lot. G1003, Wuhan, China). Finally, the images were collected and analyzed under a microscope, after dehydration and sealing. TRAP staining solution (Service bio, Lot. G1050, Wuhan, China) was pre-pared according to the instructions, and the dehydrated sections were numbered with a histochemical pen and added with TRAP staining solution. After incubation, the core was washed with water and examined by microscope after sealing. The undecalcified sections were demolded to water, and Von Kossa (VK) staining solution (Service bio, Lot. G1043, Wuhan, China) was added to the tissue immediately after circling with a histochemical pen, and then irradiated with UV lamp for 20 min.Hematoxylin staining solution and eosin staining solution were added in turn, dehydrated and sealed, followed by microscopic examination. In the Goldner experiment, dehydrated sections were placed sequentially in Goldner staining solution (Service bio, Lot. G1064, Wuhan, China), sealed and examined by microscopy.

### 2.12 Immunofluorescence (IF)

Freshly dissected bone tissue was fixed in 4% paraformaldehyde for 2 days, followed by decalcification in 10% EDTA for 2 weeks to soften the tissue for further analysis. The tissue slides were subsequently incubated at 99°C for 20 min to facilitate antigen retrieval using a citrate buffer. After cooling, the slices were incubated overnight at 4°C with the primary antibodies to ensure specific binding. The primary antibodies used in this study included CCAAT enhancer-binding protein alpha (C/EBPα) (Affinity, Lot.79n5792, Jiangsu, China), Osteocalcin (OCN) (Service bio, Lot. AC230830021, Wuhan, China), β-catenin (Affinity, Lot.50c2250, Jiangsu, China), and CD44 (Service bio, Lot. AC2308300, Wuhan, China). The following day, the slides were washed three times with PBS to remove any unbound antibodies before being incubated for one hour with the appropriate secondary antibody. Finally, images of the stained slides were captured using an Olympus IX81 microscope in order to document and analyze the results.

### 2.13 Western blot of protein

Proteins that had been preserved intact by frozen RIPA buffer (Solarbio, Lot. R0010, Beijing, China), with the integrity of the protein molecules. The lysates were electrophoretically divided based on the different mobility capabilities of proteins through the 12% SDS-PAGE polyacrylamide gel separation technique. The ablating process was followed by transferring the proteins on a 0.22 μM polyvinylidene fluoride (PVDF) membrane which was carried out in the wet transfer method. The membrane was then coated with 5% BSA for an hour at room temperature to make it less attractive for non-specific binding. After that, incubation of the membrane was done over-night at 4°C with primary antibodies having specificity to activating transcription factor 4 (ATF4) (Proteintech, Lot.10023863, Wuhan, China), β-catenin (Affinity, Lot.50c2250, Jiangsu, China), peroxisome proliferator-activated receptor gamma (PPAR-γ) (Affinity, Lot.60s1447, Jiangsu, China), beta-actin (β-actin) (Bioworld, Lot.2023090912, Beijing, China). When it was washed scrupulously the next day, it was then incubated at room temperature with a horseradish peroxidase (HRP)-conjugated secondary antibody (Elabscince, Lot. Xxf066lrv3352, Wuhan, China; Cowin Biotech, Lot.01334/00,724, Jiangsu, China) for one hour. At last, the diffusion of the antibody-name substances was made lively by makeshift luminophore creation (ECL) instrument which was used for their detection.

### 2.14 Statistical analysis

The data are depicted as the mean ± standard deviation (SD). Statistical analyses were implemented via GraphPad Prism software to warrant accurate and dependable results. To appraise statistical significance between two groups, an unpaired T-test was adopted. In all experiments, a *P*-value less than 0.05 (P < 0.05) was deemed statistically significant, signifying a notable disparity between the examined groups.

## 3 Results

### 3.1 Irisin inhibits BMSCs-derived adipogenesis and promotes osteogenesis

**FIGURE 1:**
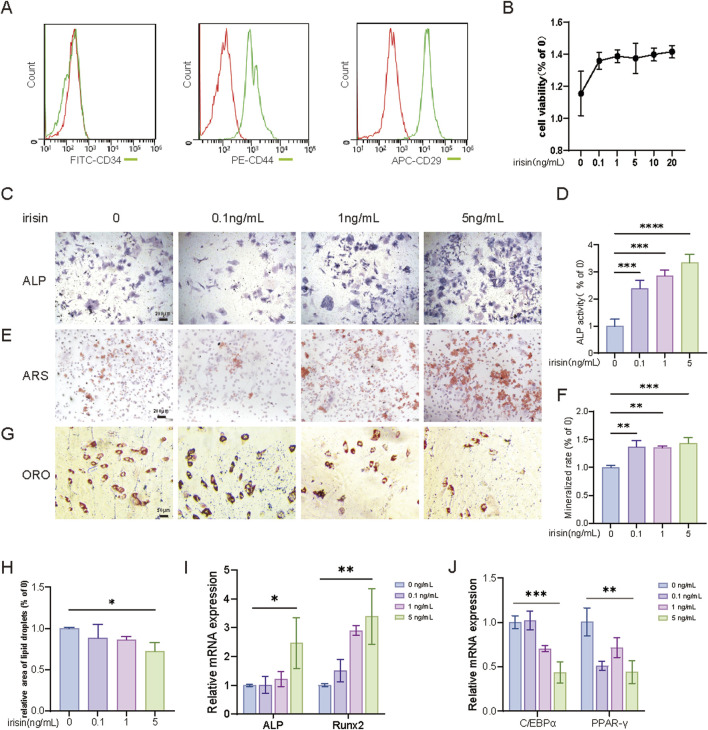
Irisin inhibits adipogenesis of BMSCs and promotes osteogenesis. **(A)** Identification of primary BMSCs cells by flow cytometry. **(B)** CCK-8 was used to detect the proliferation activity of BMSCs treated with irisin (0, 0.1, 1, 5, 10, and 20 ng/mL). **(C)** The representative images of ALP staining after treatment with irisin (0、0.1、1、5 ng/mL) for 7 days (4×). **(D)** The quantitative assessment of the ALP staining area. **(E)** The representative images of ARS staining after treatment with irisin (0、0.1、1、5 ng/mL) for 14 days (4×). **(F)** The quantitative assessment of the ARS staining area. **(G)** The representative images of Oil red O (ORO) staining after treatment with irisin (0、0.1、1、5 ng/mL) for 21 days (20×). **(H)** The quantitative assessment of the ORO staining area. **(I)** After osteogenesis for 7 days, the mRNA expression levels of Runx2, ALP in BMSCs stimulated with irisin (0, 0.1, 1, 5 ng/mL) by RT-qPCR. **(J)** After osteogenesis for 7 days, the mRNA expression levels of PPAR-γ, C/EBPα in BMSCs stimulated with irisin (0, 0.1, 1, 5 ng/mL) by RT-qPCR. (n = 3, *P < 0.05, **P < 0.01, ***P < 0.001).

To explore the specific role of irisin in the differentiation process of BMSCs, initially, stromal cells in the bone marrow cavity of mice were extracted and cultivated. The flow cytometry results indicated CD34 (−), CD44 (+), and CD29 (+) to verify the purity of BMSCs (Figure 1A). The effect of irisin on the proliferative capacity of BMSCs was evaluated through the CCK-8 assay. The results indicated that irisin at concentrations ranging from 0.1 to 20 ng/mL had no significant impact on the proliferative activity of BMSCs (Figure 1B). As illustrated in Figures 1C, D, irisin significantly enhanced the alkaline phosphatase (ALP) positive area in BMSCs in a dose-dependent manner 7 days post-osteogenic induction (P < 0.05). Alizarin red staining (ARS) revealed that irisin significantly enhanced calcium deposition in BMSCs 14 days following osteogenic induction (Figures 1E, F). Notably, the enhancement was most pronounced in the treatment group receiving 5 ng/mL of irisin (P < 0.05). After 21 days of adipogenic induction (Figures 1G, H), irisin significantly decreased the number of lipid droplets in differentiated adipocytes derived from BMSCs (P < 0.05). Quantitative real-time PCR (RT-qPCR) results (Figure 1I) revealed that the expressions of runt-related transcription factor 2 (Runx2) and ALP associated with osteogenic differentiation were augmented by 3.39 and 2.47 folds, respectively, while the expressions of peroxisome proliferator-activated receptor gamma (PPAR-γ) and CCAAT enhancer binding protein alpha (C/EBPα) related to lipogenesis were diminished by 0.47 and 0.41 folds in BMSCs treated with irisin (5 ng/mL). Considering the previous studies (Zhang et al., 2024) and the results obtained in the aforementioned experiments, a concentration of 5 ng/mL irisin was chosen for the treatment of BMSCs in subsequent experiments.

### 3.2 Irisin reverses the decline in pluripotency of senescent BMSCs

**FIGURE 2:**
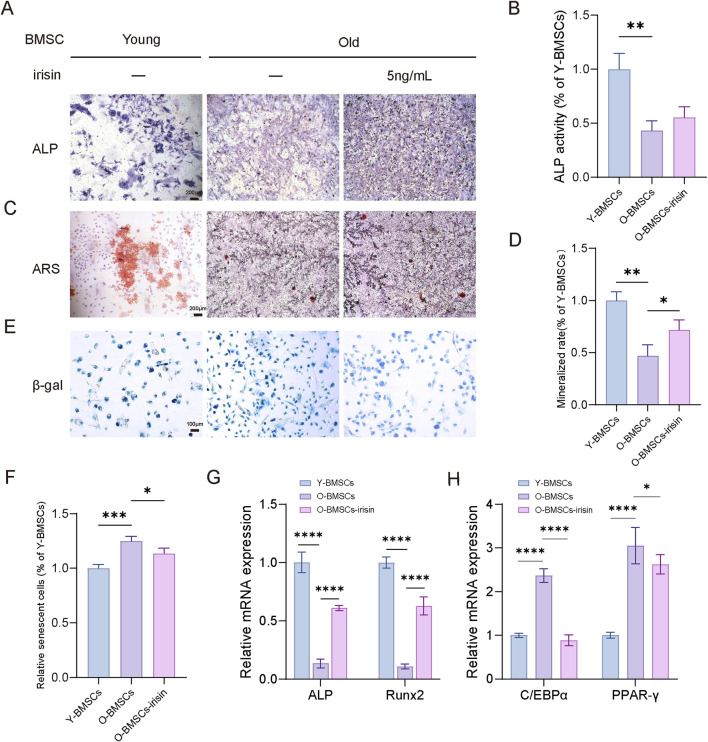
Irisin contributes to the pluripotent recovery of senescent BMSCs. **(A)** Representative image of ALP staining of young and aged BMSCs following irisin (5 ng/mL) or PBS treatment for 7 days (4×). **(B)**The quantitative assessment of the ALP staining area. **(C)** Representative image of ARS staining of young and aged BMSCs following irisin (5 ng/mL) or PBS treatment for 7 days (4×). **(D)** The quantitative assessment of the ARS staining area. **(E)** Representative image of SA-β-Gal staining of young and aged BMSCs following irisin (5 ng/mL) or PBS treatment for 7 days (10×). **(F)** The quantitative assessment of the SA-β-Gal staining area. **(G)** After osteogenesis for 7 days, the mRNA expression levels of Runx2 and ALP in BMSCs stimulated with irisin or PBS treatment by RT-qPCR. **(H)** After adipogenesis for 10 days, the mRNA expression levels of PPAR-γ and C/EBPα in BMSCs stimulated with irisin or PBS treatment by RT-qPCR. (*n* = 3, *P < 0.05, **P < 0.01, ***P < 0.001).

To investigate the effects of irisin on the self-renewal and pluripotency of aging BMSCs, BMSCs were isolated from young (2-month-old) and aged (15-month-old) male C57BL6/J mice, with or without stimulation by irisin (5 ng/mL). The results indicated (Figures 2A–D) that the osteogenic capacity of young BMSCs (Y-BMSCs) was significantly lower than that of old BMSCs (O-BMSCs). Furthermore, the ALP activity and mineralization area in O-BMSCs treated with irisin (O-BMSCs-irisin) were markedly increased (P < 0.05). As demonstrated by β-galactosidase (SA-β-Gal) staining (Figures 2E, F), the number of senescent cells was significantly elevated in the O-BMSCs group. In contrast, the positive area was notably reduced in the O-BMSCS-irisin group (P < 0.05). The results of RT-qPCR (Figures 2G, H) revealed that the mRNA expression levels of ALP and Runx2 in the O-BMSCs-irisin group were enhanced by 4.52 and 5.81 folds, respectively, in comparison with the O-BMSCs group (P < 0.05). On the contrary, PPAR-γ and C/EBPα presented decreases of 0.859 and 0.374 folds (P < 0.05). These findings suggest that irisin could, to a certain extent, enhance the self-renewal capacity and pluripotency restoration of senescent BMSCs.

### 3.3 Irisin mediates the Wnt/β-catenin pathway to regulate the pluripotency of aging BMSCs

**FIGURE 3:**
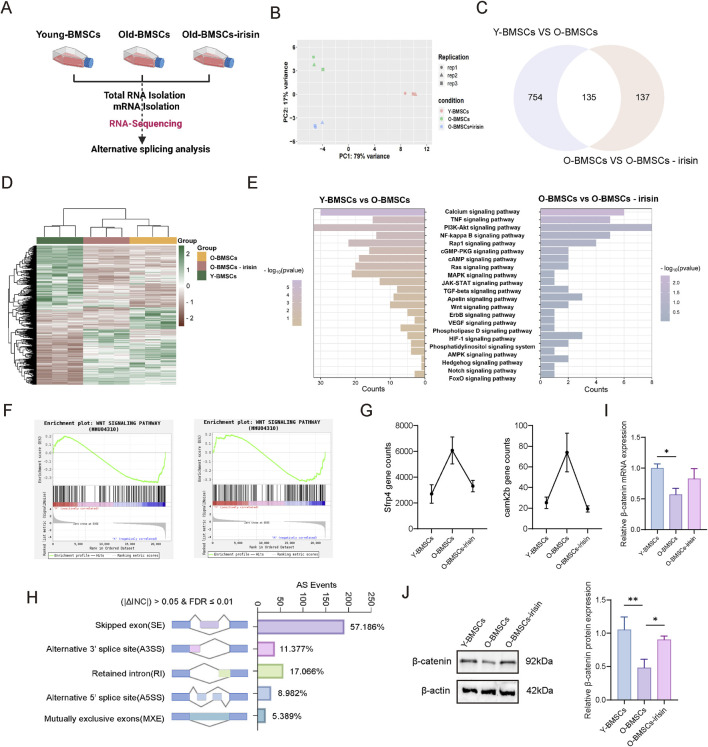
Genome-wide analysis of the regulation of pluripotency of senescent BMSCs by irisin. **(A)** Schematic diagram of RNA-sequencing experiment. **(B)** PCA cluster analysis. **(C)** Venn diagram depicting the differential genes among the three groups. **(D)** The value of relative expression abundance of DEG was analyzed through heat map. **(E)** KEGG pathway analysis, top 10 significantly enriched pathways (−log10 (*p*-value < 0.05)). **(F)** GSEA of Wnt signaling pathways. **(G)** Relative expression levels of the Camk2b and Sfrp4 genes in the downstream signaling of the Wnt pathway. **(H)** Comparison between the O-BMSCs group and the O-BMSCs-irisin group, presented as a histogram of variable shear events and alternative splicing variations. **(I)** mRNA expression levels of β-catenin gene were detected by RT-qPCR. **(J)** Characteristic images and quantitative analysis of β-catenin relative protein expression levels. (*n* = 3, *P < 0.05, **P < 0.01, ***P < 0.001).

Next, the underlying mechanisms by which irisin regulates the pluripotency and differentiation potential of senescent BMSCs were explored through RNA-sequence and alternative splicing analysis (Figure 3A). Principal Component Analysis (PCA) cluster analysis (Figure 3B) showed significant differences among the three groups. There were 889 differentially expressed genes (DEGs) identified between the Y-BMSCs group and the O-BMSCs group. Additionally, 272 DEGs were detected between the O-BMSCs group and the O-BMSCS-irisin group. Notably, there were 135 common DEGs shared between these two groups, which facilitated the generation of a heat map (Figures 3C, D). The Kyoto Encyclopedia of Genes and Genomes (KEGG) analysis indicated (Figure 3E) that the addition of irisin was significantly associated with various signaling pathways, including the PI3K-Akt, NF-κB, Wnt, Hedgehog, and Notch pathways. Subsequently, gene set enrichment analysis (GSEA) revealed that irisin was closely associated with the Wnt pathway and downregulated the expression of downstream target genes, specifically Camk2b and Sfrp4 (Figures 3F, G). Several types of differentially variable shear events in the O-BMSCs and O-BMSCS-irisin groups were analyzed, and their percentages were computed (|ΔINC| > 0.05, FDR ≤0.01). Pre-mRNA splicing (Figure 3H) showed changes in alternative 50 splice sites (8.982%), exon skipping (57.186%), intron retention (17.066%), mutually exclusive exons (5.389%), and alternative 30 splice sites (11.377%). Among them, variable shear was observed at the β-catenin shear site; however, this occurrence was not statistically significant. Western blot and RT-qPCR analyses demonstrated (Figures 3I, J) that the expression levels of both β-catenin protein and mRNA were significantly elevated in the O-BMSCs-irisin group compared to the O-BMSCs group (P < 0.05). Alterations in splicing factors and pre-mRNA may significantly contribute to the functional decline of BMSCs as they age.

### 3.4 Irisin modulates the β-catenin/PPAR-γ/ATF4 signaling pathway to influence the osteogenic and adipogenic differentiation of BMSCs

**FIGURE 4:**
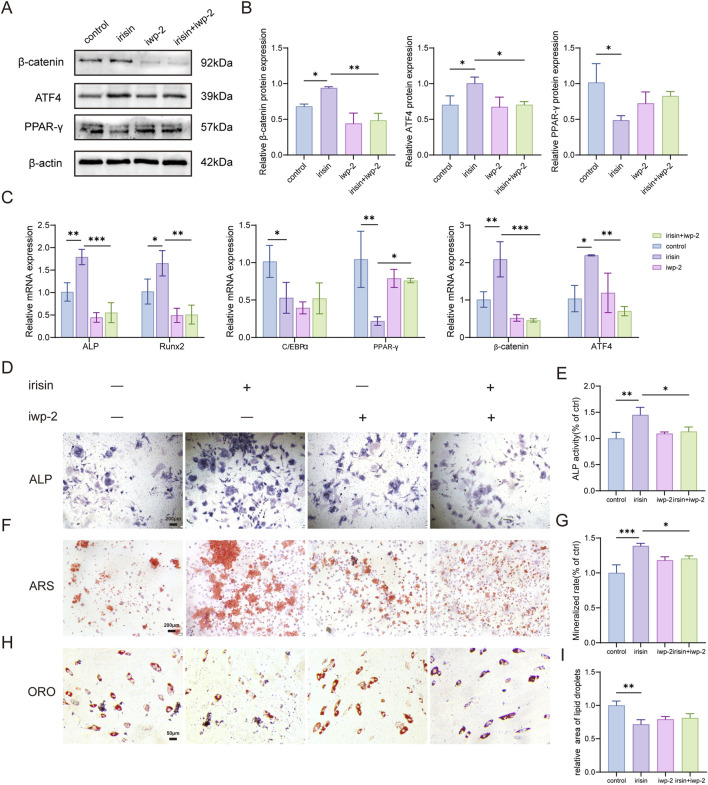
Irisin enhances osteogenesis and improves adipogenesis through the activation of Wnt signaling pathways. **(A)** Characteristic image of the relative protein expression levels of β-catenin. **(B)** Quantitative analysis of β-catenin protein. **(C)** The expression levels of Runx2, ALP, PPAR-γ, C/EBPα, ATF4, and β-catenin mRNA in BMSCs stimulated by iwp-2 with or without the combination of irisin were analyzed using RT-qPCR. **(D)** Representative images of ALP staining in BMSCs treated with either irisin (5 ng/mL) or iwp-2 (5 μM). **(E)** The quantitative assessment of the ALP staining area. **(F)** Representative images of ARS staining in BMSCs treated with either irisin (5 ng/mL) or iwp-2 (5 μM). **(G)** The quantitative assessment of the ARS staining area. **(H)** Representative images of ORO staining in BMSCs treated with either irisin (5 ng/mL) or iwp-2 (5 μM). **(I)** The quantitative assessment of the ORO staining area. (*n* = 3, *P < 0.05, **P < 0.01, ***P < 0.001.).

To further investigate the significance of the Wnt signaling pathway in the regulation of irisin on BMSCs differentiation, iwp-2 (a potent antagonist of the Wnt pathway) was administered either alone or in conjunction with irisin. The results demonstrated (Figures 4A, B) that the expression of β-catenin and downstream activating transcription factor 4 (ATF4) protein in BMSCs treated with irisin was augmented, and the expression of PPAR-γ protein was reduced (P < 0.05). However, treatment with iwp-2 resulted in a significant decrease in the protein levels of β-catenin and ATF4, while concurrently increasing the protein content of PPAR-γ. Similarly, RT-qPCR analysis demonstrated (Figure 4C) that irisin significantly upregulated the expression of β-catenin, ATF4, and osteogenic genes such as Runx2 and ALP, while levels of PPAR-γ and C/EBPα were found to be decreased (P < 0.05). In combination with iwp-2, this effect was reversed (P < 0.05). Additionally, as shown in Figures 4D–I, irisin stimulation led to a significant increase in ALP expression and mineralized area, along with a reduction in lipid droplet formation. However, the introduction of iwp-2 attenuated these effects (P < 0.05). These findings indicate that irisin may play a role in the processes of bone formation and adipogenesis in BMSCs via the Wnt signaling pathway. Furthermore, the underlying mechanism appears to be associated with the reliance on downstream target genes ATF4 and PPAR-γ.

### 3.5 Irisin alleviates the bone mass reduction associated with aging imageologically

**FIGURE 5:**
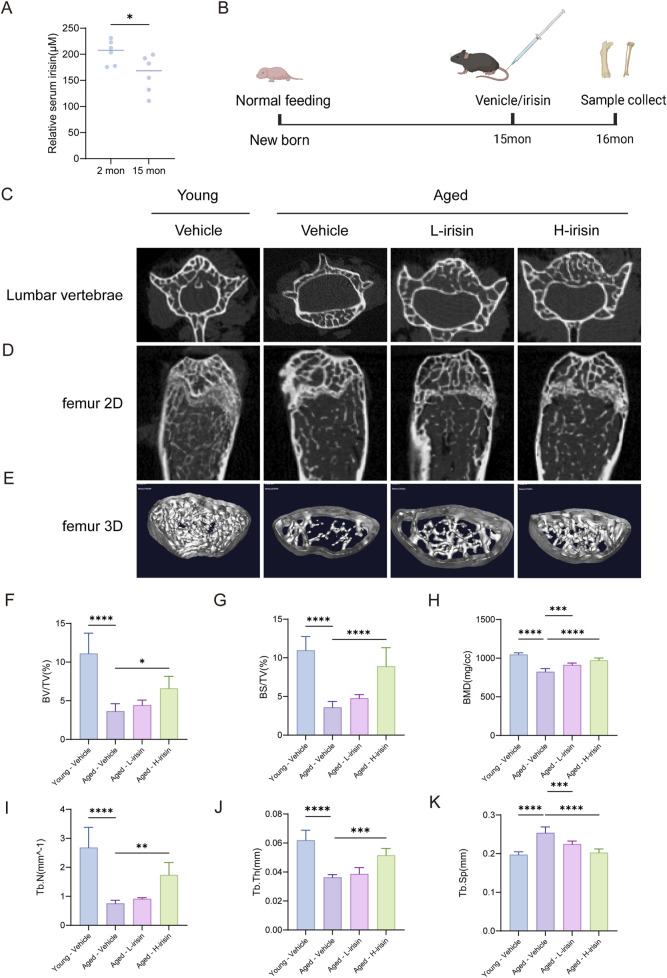
Irisin alleviates age-related declines in bone mass as observed through imaging studies. **(A)** Analysis of serum irisin levels in mice. **(B)** Schematic diagram of animal experiments. **(C)** Representative image of the plain scan reconstructed from micro-CT of the lumbar vertebra 4 (L4) in mice. **(D)** Coronal plane scan of the distal femur in mice reconstructed using micro-CT. **(E)** Three-dimensional layered imaging of the distal femoral diaphysis in mice. **(F)** Quantitative analysis of bone mineral density (BMD, mg/cc) in trabecular bone. **(G)** Comparative analysis of bone volume (BV/TV, %). **(H)** Comparative analysis of trabecular area and volume (BS/TV, %). **(I)** Quantitative analysis of trabecular thickness (Tb. Th, mm). **(J)** Quantitative analysis of trabecular number (Tb. N, 1/mm). **(K)** Quantitative analysis of trabecular spacing (Tb. Sp, mm). (*n* = 6, *P < 0.05, **P < 0.01, ***P < 0.001).

The following study investigated the effects of irisin on a mice model of osteoporosis under aging conditions. The levels of irisin were assessed in young (2-month-old) and aged (15-month-old) male C57BL6/J mice (Figure 5A). It was observed that the levels of irisin were significantly reduced in aged mice compared to their younger counterparts (P < 0.05). Subsequently, young mice were administered a Vehicle (Young-Vehicle), while aged mice received either a Vehicle (Aged-Vehicle), 25 mg/kg of irisin (Aged-L-irisin), or 100 mg/kg of irisin (Aged-H-irisin) (Figure 5B). Micro CT analysis was conducted to evaluate the changes in the microstructural properties of femurs and vertebrae in mice. As illustrated in Figures 5C–E, compared to the Young-Vehicle group, the Aged-Vehicle group exhibited significant structural damage in vertebrae. The trabecular architecture of the femurs appeared notably sparse and fragmented, with pronounced fractures and conspicuous bone marrow cavities. After treatment with irisin, the analysis of microstructural bone parameters (Figures 5F–K) revealed varying degrees of increase in bone mineral density (BMD), bone volume fraction (BV/TV), bone surface fraction (BS/TV), trabecular thickness (Tb. Th), and trabecular number (Tb. N). Notably, the Aged-H-irisin group exhibited a more pronounced rise in these parameters (P < 0.05). In contrast, trabecular separation distance (Tb. Sp) showed a significant decrease (P < 0.05).

### 3.6 Irisin promotes the restoration of skeletal health histologically in aging individuals

**FIGURE 6:**
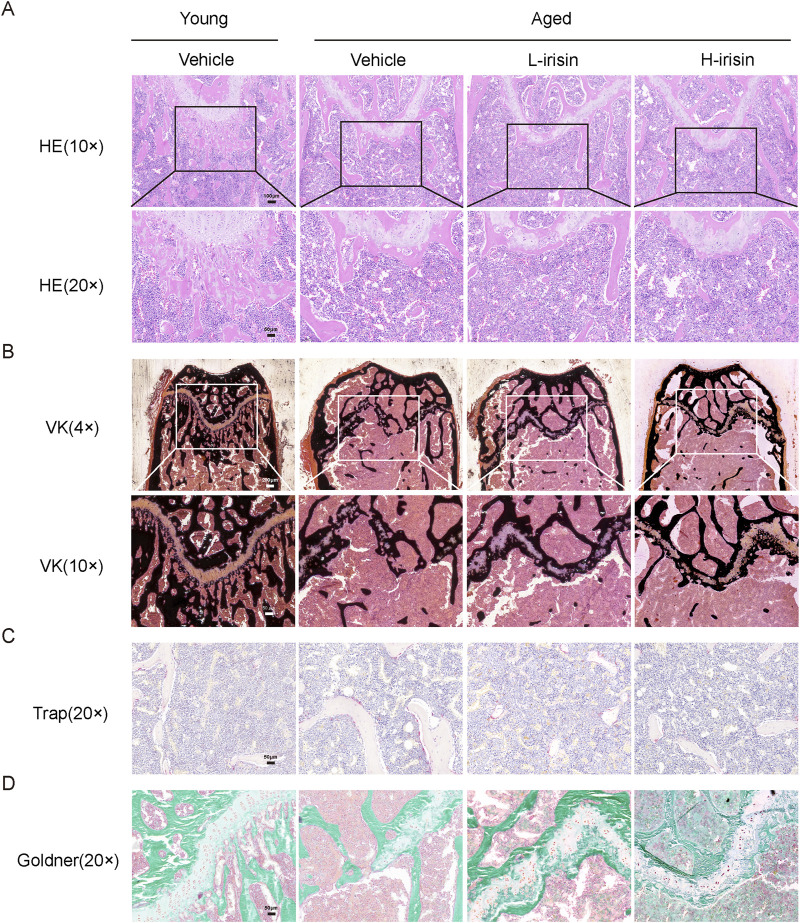
Irisin promotes the restoration of skeletal health in aging morphology. **(A)** Representative HE staining images of the femur (10× and 20×). **(B)** Representative VK staining images of the femur (4× and 10×). **(C)** Representative TRAP staining images of the femur (20×). **(D)** Representative Goldner staining images of the femur (20×).

The pathological morphology observed is similar to that seen in Micro CT. H&E staining results (Figure 6A) indicate that, compared to the Young-Vehicle group, the Aged-Vehicle group exhibits a more sparse and thinner bone structure in the lower segment of the growth plate, accompanied by the formation of lipid droplets. After the injection of irisin, there was a notable reduction in the bone marrow adipose tissue within the medullary cavity. Von Kossa (VK) staining indicated (Figure 6B) that the growth plate fractures in the Aged-Vehicle group were significantly pronounced, accompanied by a marked decrease in the area of mineralized bone. The administration of irisin has partially reshaped the formation of the growth plate, thereby promoting bone mass recovery to a certain extent. TRAP staining reveals (Figure 6C) that there is a significant increase in osteoclasts in the Aged-Vehicle group, indicating a marked elevation in bone resorption levels. Furthermore, Goldner staining (Figure 6D) revealed significant destruction of cortical collagen bone in the Aged-Vehicle group. However, administration of irisin resulted in an improvement in osteoid formation. In summary, iris extract has the potential to mitigate bone damage in the femur and vertebrae of aging mice, thereby promoting skeletal health.

### 3.7 Irisin alleviates the reduction of bone formation and the increase of fat in aging mice

**FIGURE 7:**
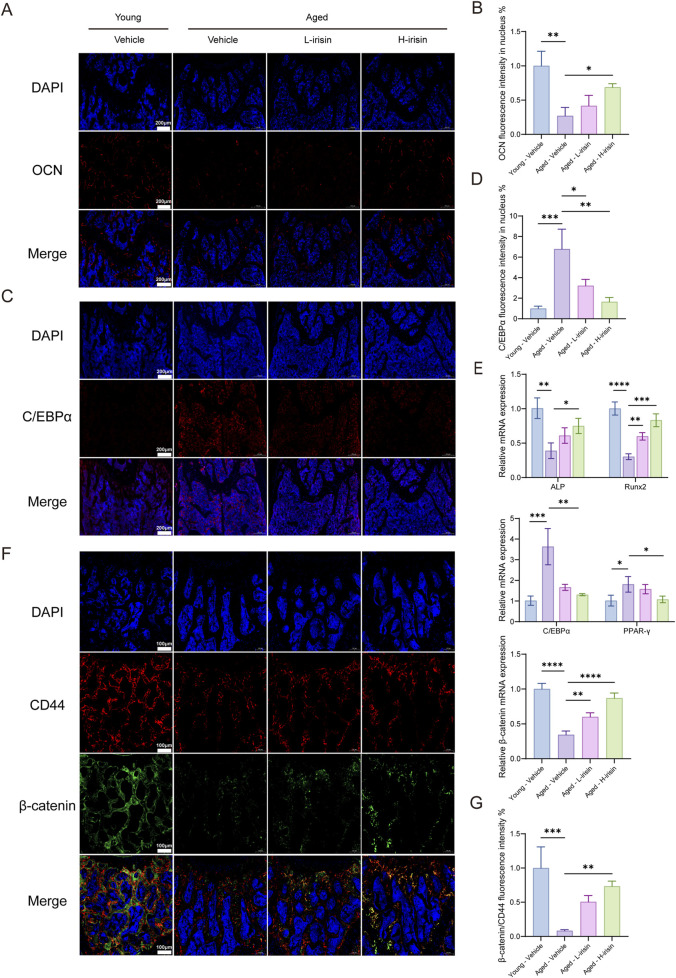
Irisin improved bone formation reduction and fat increase in aging mice. **(A)** Representative fluorescent images of OCN in mice femur tissue. **(B)** Quantitative analysis of OCN fluorescence. **(C)** Representative fluorescence images of C/EBPα in mice femur tissue. **(D)** Quantitative analysis of C/EBPα fluorescence. **(E)** The RT-qPCR method was employed to assess the mRNA expression levels of ALP, Runx2, C/EBPα, PPAR-γ, and β-catenin genes. **(F)** Representative fluorescence images of double staining for β-catenin and CD44 in mouse femoral tissue. **(G)** Quantitative analysis of fluorescence of β-catenin relative to CD44. (*n* = 3, *P < 0.05, **P < 0.01, ***P < 0.001).

In order to investigate the effects of irisin on osteogenesis and adipogenesis in aging bones, we employed fluorescent staining to localize and label proteins associated with bone-fat formation. The immunofluorescence results (Figures 7A–D) indicate that, compared to the Young-Vehicle group, the Aged-Vehicle group exhibited a significant decrease in fluorescence intensity of the bone gene OCN, while there was a notable increase in the expression of C/EBPα (P < 0.05). After treatment with irisin, the observed alterations were reversed. The RT-qPCR (Figure 7E) results demonstrated that diverse doses of irisin significantly augmented the mRNA levels of ALP and Runx2 in bone tissue, while the expression of PPAR-γ and C/EBPα declined. On the contrary, the expression of β-catenin was found to be elevated (P < 0.05). Subsequently, the localization and expression of β-catenin in the femur were verified. It was noted that β-catenin showed significant colocalization with the BMSC marker protein CD44 (Figures 7F, G). The findings suggest that irisin is capable of facilitating bone remodeling in aged bones and diminishing the formation of bone marrow adipose tissue. This effect might be related to the Wnt signaling pathway.

## 4 Discussion

The theory of stem cells plays a crucial role in regulating bone homeostasis within biological organisms, encompassing the transformation and fate determination of both osteogenic and adipogenic lineages (Xiao et al., 2023). As aging advances, the functionality of BMSCs deteriorates, resulting in a reduced self-renewal capacity and a gradual loss of multipotency (Xu et al., 2024). This disturbance in the homeostasis of the bone marrow microenvironment can give rise to age-related osteoporosis (Ge et al., 2017). Additional evidence indicates that during physical activity, skeletal muscle secretes numerous myokines that are involved in endocrine and paracrine processes within the bone marrow microenvironment, contributing to the metabolic reprogramming indispensable for maintaining skeletal health (Schumm et al., 2023). However, older adults frequently demonstrate significantly insufficient levels and duration of physical exercise. In view of this situation, we propose exploring a novel myokine as an alternative therapeutic approach to exercise intervention.

In recent years, it has been affirmed that irisin exhibits certain bone-protective effects (Zhu et al., 2023). Irisin is discharged during skeletal muscle contraction and enhances the expression of peroxisome proliferator-activated receptor γ coactivator 1-α (PGC-1α), thereby resulting in the proteolytic cleavage of the N-terminal region of the transmembrane protein FNDC5 (Lee et al., 2021). Research has indicated that silencing or overexpressing the irisin receptor integrin αVβ5 can have an impact on the fate determination and differentiation direction of BMSCs (Zhu et al., 2023). This study further extends the application scope of irisin in ameliorating age-related metabolic disorders through the utilization of cellular models. *In vitro*, irisin participates in the pluripotency of BMSCs in a dose-dependent manner, promoting osteogenesis and attenuating adipogenesis. Subsequently, a natural aging model of BMSCs was established, uncovering distinct aging characteristics in senescent BMSCs. The stemness and self-renewal capacity were significantly reduced, and compared with normal mouse BMSCs, there was a heightened tendency towards adipogenic differentiation. This finding is in line with previous research conclusions (Farr et al., 2017). Moreover, the addition of irisin partially alleviated the aging rate of BMSCs and restored their pluripotent capabilities, while promoting osteogenic differentiation.

The Wnt signaling pathway has been recognized as a crucial signaling cascade for the prevention and treatment of osteoporosis. Reports suggest that irisin can mediate osteoblast proliferation and mineralization and regulate osteoclast differentiation and maturation via the Wnt signaling cascade (Hu et al., 2024; Sheng et al., 2023; Waseem et al., 2022). Significantly, the Wnt signaling pathway constitutes a key element in irisin’s facilitation of osteogenic differentiation in BMSCs (Chen et al., 2020). Based on this comprehension, we further probed into the potential mechanisms through which irisin governs the fate determination of aging BMSCs. RNA sequencing disclosed that the Wnt pathway was prominently enriched among the top 20 pathways. GSEA indicated that Irisin might alleviate the decline in stemness of BMSCs by upregulating the Wnt pathway. Moreover, abnormal alternative splicing can give rise to cellular senescence and exert an influence on differentiation pathways (Aprile et al., 2018; Maier et al., 2004). It has been documented that BMSCs from donors of different ages display distinct selective splicing events (Peffers et al., 2016). We analyzed the potential alternative splicing elicited by irisin in aging BMSCs, and identified the involvement of the Wnt family. Subsequently, we carried out *in vitro* validation and discovered that irisin upregulated β-catenin levels in aging BMSCs.

The research reveals that the Wnt/β-catenin signaling pathway is one of the vital constituents implicated in the process of cellular adipocyte differentiation (Takada et al., 2009; Yuan et al., 2016). The classical Wnt/β-catenin pathway is capable of suppressing the expression of PPAR-γ mRNA, while the non-classical Wnt pathway activates histone methyltransferases, which inhibit the transactivation of PPAR-γ by mediating the methylation of lysine 9 on histone H3 (H3K9) at its target genes (Takada et al., 2009). On this basis, we combined irisin with the Wnt inhibitor iwp-2 and observed a reduction in the inhibitory effect of irisin on PPAR-γ activation, as well as a decrease in its suppression of adipogenesis. Nevertheless, this study has certain limitations. Specifically, we did not carry out further validation using gene-edited mice at the animal level. Additionally, as the mechanism of action of irisin remains unclear, it might directly engage in the transcriptional regulation of adipogenic differentiation of BMSCs as a nuclear factor or function as a cytokine exerting autocrine or paracrine effects; this demands further exploration.

**FIGURE 8:**
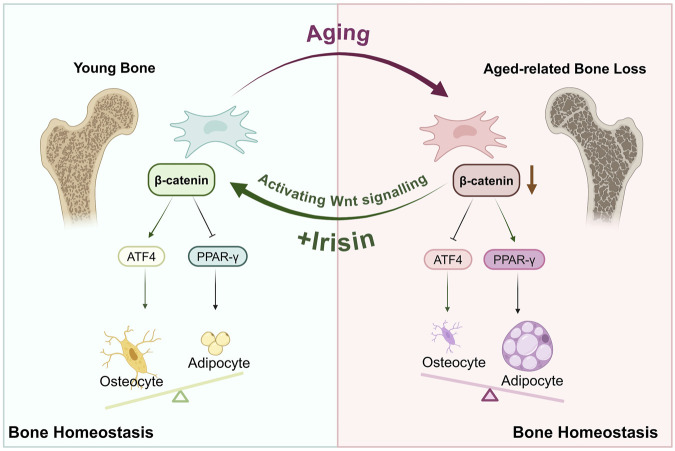
Irisin orchestrates the engagement of Wnt/β-catenin/ATF4/PPAR-γ in the osteogenic and adipogenic differentiation of BMSCs within the bone marrow microenvironment.

ATF4 is a member of the DNA-binding protein family, which includes the AP-1 transcription factor family and cAMP response element-binding protein (CREB), among others (Wang et al., 2012). Research has indicated that the transcriptional activity of ATF4 increases during the differentiation of BMSCs into osteoblasts (Feng et al., 2022). Furthermore, inhibition of β-catenin can block ATF4-mediated osteogenic differentiation and proliferation of BMSCs (Hu et al., 2023). Interestingly, further investigations (Shen et al., 2021) have revealed that Wnt signaling enhances glutamine uptake during osteoblast proliferation; ATF4 serves as a critical mediator for Wnt-induced expression of Slc1a5. Inhibition of ATF4 results in decreased levels of Slc1a5 and subsequently impairs glutamine uptake, which adversely affects bone formation. This study found that irisin can promote ATF4 expression, thereby enhancing osteogenic differentiation. However, following the administration of iwp-2, ATF4 expression decreased, indicating that irisin may enhance β-catenin expression to subsequently elevate downstream ATF4 levels and exert its osteogenic effects. Collectively, these results suggest that PPAR-γ and ATF4 may serve as crucial indicators of the anti-osteoporotic effects of irisin; further research is warranted (Figure 8).

In current research, irisin has been utilized in diverse osteoporosis models, such as bilateral ovariectomy, obesity, and type 1 diabetes mouse models. It not only boosts the proliferation of osteoblasts and inhibits ferroptosis but also lowers the expression of IL-6 in adipocytes. These discoveries altogether suggest its anti-osteoporotic effects (Storlino et al., 2023; Zhang et al., 2024; Mohsin et al., 2023). Moreover, it has been discovered that irisin is implicated in the advancement of various age-related disorders (Fatouros, 2018; McPhee et al., 2016), such as obesity, diabetes, and sarcopenia—complexities associated with aging (de Freitas et al., 2020; Guo et al., 2023; Marrano et al., 2021). It holds the potential to alleviate oxidative stress and dampen inflammatory responses, thereby facilitating the reversal of aging processes. The current study refers to previous investigations and employs naturally aged C57BL/6J mice to establish an aging model. It was noted that the serum irisin levels in aged mice were conspicuously reduced. Morphological and imaging evaluations disclosed a notable decline in bone mass and a considerable disruption of bone microstructure in aged mice when compared with younger ones. Significantly, intervention with irisin resulted in a considerable increase in bone mass, facilitated bone remodeling, and decreased adipogenesis. Additionally, the dual localization of β-catenin and CD44 affirmed that irisin upregulates the expression of β-catenin in BMSCs. This targeted delivery of myokines significantly enhanced both the safety and efficacy of anti-osteoporosis treatment *in vivo*.

## 5 Conclusion

In conclusion, this study recognizes irisin as a myokine capable of participating in the age-related lineage commitment of BMSCs through the activation of the Wnt/β-catenin/ATF4/PPAR-γ signaling cascade. Additionally, it enhances self-renewal and stemness restoration in aging BMSCs, thus maintaining the equilibrium of the bone marrow microenvironment.

## Data Availability

The datasets presented in this study can be found in online repositories. The names of the repository/repositories and accession number(s) can be found below: NCBI repository (https://www.ncbi.nlm.nih.gov/), accession number PRJNA1182913.
